# Supplementary material for: A multi-domain collaborative denoising bearing fault diagnosis model based on dynamic inter-domain attention mechanism and noise-aware loss function
Source: PLoS One. 2025 Jun 26;20(6):e0326666. doi: 10.1371/journal.pone.0326666 (PMC12200651; doi:10.1371/journal.pone.0326666)
Supplement: Attachment — Submitted filename: Response to Reviewers.docx [file pone.0326666.s001.docx]

**Original Manuscript ID:** PONE-D-25-14574

**Original Article Title:** “A Multi-Domain Collaborative Denoising Bearing Fault Diagnosis Model Based on Dynamic Inter-Domain Attention Mechanism and Noise-Aware Loss Function”

Dear Academic Editor,

Hello,

Firstly, we would like to express our sincere gratitude to you and the peer-review team for the valuable feedback and meticulous review of our manuscript. Second, we have carefully considered the journal requirements and reviewers' comments and suggestions and made corresponding revisions to the full text to improve its quality and clarity. Finally, we believe these revisions will strengthen the academic quality and practicality of the manuscript. Thank you for your thoughtful guidance. We will continue to strive to meet the high standards of the journal.

We have submitted the revised manuscript and a document detailing our point-by-point response to the journal requirements and reviewers' comments. We kindly ask the reviewers and the editorial team to review the revisions, and we look forward to receiving further guidance and suggestions.

Once again, we sincerely appreciate your time and effort, as well as your support and valuable feedback on our research. We look forward to your final review and hope for your approval.

Best regards,

<Weilin Cao> et al.

**Journal requirements, Concern#1:** Please ensure that your manuscript meets PLOS ONE's style requirements, including those for file naming. The PLOS ONE style templates can be found at

https://journals.plos.org/plosone/s/file?id=wjVg/PLOSOne_formatting_sample_main_body.pdf and

https://journals.plos.org/plosone/s/file?id=ba62/PLOSOne_formatting_sample_title_authors_affiliations.pdf.

**Author response:** We sincerely thank the editorial office for the reminder regarding the manuscript formatting requirements. In response to this concern, we have carefully reviewed the relevant guidelines on the PLOS ONE official website. Our manuscript was prepared using the LaTeX template available under the path: **Publish → Submission Guidelines → File Format → Read the LaTeX guidelines → Download the template** (<https://journals.plos.org/plosone/s/latex>). We have made every effort to ensure that the formatting aligns with the official template. We would greatly appreciate further instructions if there are any specific discrepancies we may have missed and will be happy to make the necessary adjustments.

**Journal requirements, Concern#2:** Please note that PLOS ONE has specific guidelines on code sharing for submissions in which author-generated code underpins the findings in the manuscript. In these cases, we expect all author-generated code to be made available without restrictions upon publication of the work. Please review our guidelines at https://journals.plos.org/plosone/s/materials-and-software-sharing#loc-sharing-code and ensure that your code is shared in a way that follows best practice and facilitates reproducibility and reuse.

**Author response:** We sincerely thank the editorial office for the reminder regarding the code-sharing policy. We fully acknowledge and respect PLOS ONE’s emphasis on transparency and reproducibility in scientific research. However, we regret to inform you that the code developed for this study cannot be made publicly available due to institutional confidentiality agreements. The code is part of a project conducted under our institution’s internal regulations, prohibiting the public release of source code without prior approval.

We greatly appreciate your understanding, and we have done our best to describe the methodology in sufficient detail in the manuscript to ensure the reproducibility of our results. Should the editorial office require further clarification on specific technical aspects, we would happily provide additional information where possible.

**Journal requirements, Concern#3:** Thank you for stating the following financial disclosure:

“This research was supported by Research and Innovation Team Project of Neijiang Normal University (No. 18TD02).”

Please state what role the funders took in the study. If the funders had no role, please state: "The funders had no role in study design, data collection and analysis, decision to publish, or preparation of the manuscript."

If this statement is not correct you must amend it as needed.

Please include this amended Role of Funder statement in your cover letter; we will change the online submission form on your behalf.

**Author response:** We sincerely thank the editorial office for the guidance regarding the financial disclosure and the Role of the Funder statement. Following the journal’s requirements, we confirm that:

“The funders had no role in study design, data collection and analysis, decision to publish, or preparation of the manuscript.”

We have included this statement in the revised cover letter, as requested. Thank you for your help updating the online submission form accordingly.

**Journal requirements, Concern#4:** Thank you for stating the following in the Acknowledgments Section of your manuscript:

“This research was supported by Research and Innovation Team Project of Neijiang Normal University (No. 18TD02).”

We note that you have provided funding information that is currently declared in your Funding Statement. However, funding information should not appear in the Acknowledgments section or other areas of your manuscript. We will only publish funding information present in the Funding Statement section of the online submission form.

Please remove any funding-related text from the manuscript and let us know how you would like to update your Funding Statement. Currently, your Funding Statement reads as follows:

“This research was supported by Research and Innovation Team Project of Neijiang Normal University (No. 18TD02).”

Please include your amended statements within your cover letter; we will change the online submission form on your behalf.

**Author response:** We sincerely thank the editorial office for clarifying the placement of funding information. In response to your suggestion, we have already removed the funding-related text from the Acknowledgments section of the manuscript, as requested. The amended Funding Statement has been included in the revised cover letter, as per your instructions, which now reads:

“This research was supported by the Research and Innovation Team Project of Neijiang Normal University (No. 18TD02). We confirm that the funders had no role in study design, data collection and analysis, decision to publish, or preparation of the manuscript.”

We appreciate your assistance in updating the online submission form accordingly. Thank you once again for your attention to detail.

**Reviewer#1, Concern#1:** The Abstract could benefit from a clearer emphasis on the practical significance of this research. Highlighting the engineering context and potential real-world applications would help readers better understand the value of the proposed method.

**Author response:** We sincerely appreciate your valuable suggestion regarding the abstract. Following your advice, we have carefully revised the abstract to discuss the engineering background and real-world application scenarios of rolling bearing fault diagnosis and explicitly highlight the practical significance of our work.

We believe the revised abstract better conveys the relevance of engineering and the potential practical impact of our research. The updated abstract is provided below for your reference:

*“Rolling bearings are the core transmission components of large-scale rotating machinery such as wind power gearboxes and aviation engines, so timely and effective monitoring and diagnosis of their status are crucial to ensure the stable operation of equipment, reduce maintenance costs, and improve production efficiency. However, the noise interference in the industrial field often hides the original characteristics of the bearing fault signal, leading to the deep learning-based fault diagnosis model's lack of diagnostic reliability in the strong industrial noise background. To address this problem, this paper proposes a multi-domain collaborative denoising diagnostic model based on dynamic inter-domain attention mechanism and noise-aware loss function. First, the model extracts high-dimensional features of bearing fault signals from multiple domains, such as time and frequency domains, aiming to enhance the richness and diversity of high-dimensional features to effectively suppress noise interference on the diagnostic results. Second, the dynamic inter-domain attention mechanism (DIDAM) is proposed, aiming to distinguish the importance of information in different signal domains and flexibly integrate them to realize more efficient and accurate multi-domain information fusion. Finally, the noise-aware loss function (NALF) is designed to avoid the phenomenon of the conduction model being prone to making wrong decisions due to excessive noise. Experimental results on two publicly available datasets, CWRU and MFPT, show that even in the extreme noise environment with SNR = -10 dB, the proposed model still achieves 81.25% and 76.36% fault diagnosis accuracies, which are better than most existing mainstream denoising models. Overall, the proposed method can still perform well under substantial noise interference, providing a new idea for intelligent bearing fault diagnosis in real industrial scenarios.”*

**Reviewer#1, Concern#2:** While the authors have provided a thorough review of current research, it would be beneficial to more explicitly summarize the existing research gaps before introducing the contributions and novelty of this work. Additionally, expanding the discussion to include emerging trends in machine learning and signal processing—particularly in industrial applications—could strengthen the manuscript. For example, exploring connections to recent advancements in areas such as digital twin methodology for vibration-based monitoring and prediction of gear wear, novel cyclic-correntropy based indicator for gear wear monitoring, and neuro-fuzzy system-guided cross-modal zero-sample diagnostic framework using multi-source heterogeneous non-contact sensing data could provide valuable context and demonstrate the broader relevance of this research.

**Author response:** We sincerely thank you for the insightful comments. In response to the suggestion, we have carefully revised the manuscript to more explicitly highlight existing research gaps and emphasize the novelty of our work.

Specifically, we have expanded the second and third paragraphs of the “Introduction” to discuss emerging trends in machine learning and signal processing techniques for fault diagnosis. Given the increasing dominance of deep learning approaches in recent literature, the number of publications focusing on classical signal processing or conventional machine learning methods is relatively limited. So, fewer relevant and recent studies are included; here are the references:

*[7] Wang S, Wang Q, Xiao Y, et al. Research on rotor system fault diagnosis method based on vibration signal feature vector transfer learning[J]. Engineering failure analysis, 2022, 139: 106424.*

*[8] Salunkhe V G, Khot S M, Desavale R G, et al. Unbalance Bearing Fault Identification Using Highly Accurate Hilbert–Huang Transform Approach[J]. Journal of Nondestructive Evaluation, Diagnostics and Prognostics of Engineering Systems, 2023, 6(3).*

*[9] THAMBA N B, Aravind A, Rakesh A, et al. Application of EMD, ANN and DNN for self-aligning bearing fault diagnosis[J]. Archives of acoustics, 2018, 43(2): 163–175.*

*[10] Goyal D, Dhami S S, Pabla B S. Non-contact fault diagnosis of bearings in machine learning environment[J]. IEEE Sensors Journal, 2020, 20(9): 4816-4823.*

Furthermore, in the seventh paragraph of the “Introduction” and a newly added “Discussion” section, we now clearly summarize the distinctions between our proposed approach and existing methods, highlighting the methodological innovations and practical implications.

We hope these revisions have effectively addressed your concerns and improved the clarity and relevance of our contributions.

**Reviewer#1, Concern#3:** The resolution of the figures in the manuscript could be improved to ensure clarity and enhance the overall presentation of the results.

**Author response:** We appreciate your suggestion regarding figure clarity. We want to clarify that the figures in the initially submitted manuscript were prepared and uploaded in high resolution. As shown below:

Fig.1 The overall framework of the Squeeze-and-Excitation Attention Mechanism.

Fig.2 The overall framework of the proposed multi-domain collaborative denoising bearing fault diagnosis model.

However, it is possible that the resolution was affected during the journal’s peer-review processing or PDF rendering. We will continue to pay close attention to the quality of the figures and, if necessary, re-upload higher-resolution versions to the journal’s requirements at the final submission stage. We sincerely thank you for this helpful comment.

**Reviewer#1, Concern#4:** There are occasional grammatical errors throughout the manuscript. A thorough proofreading to address these issues would improve the readability and professionalism of the paper.

**Author response:** We sincerely thank you for pointing out the grammatical issues. In response to this helpful suggestion, we have carefully proofread the entire manuscript and revised the relevant parts to improve the paper’s grammar, readability, and overall professionalism.

The revisions involve numerous small changes throughout the text, so we did not highlight each grammatical correction. Therefore, these changes are not explicitly marked in the ***Revised Manuscript with Track Changes*** file either. We kindly ask for your understanding in this regard, and we appreciate the feedback, which helped us further enhance the quality of the manuscript.

**Reviewer#1, Concern#5:** Including a section on potential future research directions at the end of the Conclusion would provide a forward-looking perspective and inspire further exploration in this field.

**Author response:** We sincerely appreciate your thoughtful suggestion. In the revised manuscript, we have added a “Discussion” section to address this point, discussing several potential directions for future research. We hope this addition enriches the manuscript and provides readers with valuable insights for further study.

**Reviewer#2, Concern#1:** The manuscript is generally clear and conveys the research content and methodology effectively. However, there are several grammatical errors, overly complex sentence structures, and awkward phrasing that affect readability. Some technical expressions are rigid, making it difficult for readers to follow the core ideas. I recommend further language polishing to enhance fluency and readability, ensuring that the content is easily understandable.

**Author response:** We sincerely appreciate your thoughtful comments and suggestions regarding our manuscript's language quality and readability. We have carefully re-read the entire manuscript and made comprehensive revisions to improve the grammar, simplify complex sentence structures, and enhance clarity and fluency.

The revisions involve numerous small changes throughout the text, so we did not highlight each grammatical correction. Therefore, these changes are not explicitly marked in the ***Revised Manuscript with Track Changes*** file either. We hope the revised version meets your expectations and is now more accessible and easier to follow.

**Reviewer#2, Concern#2:** Your study presents certain improvements and new approaches, particularly in signal processing and fault diagnosis within the field of mechatronic engineering. However, the level of innovation is not sufficiently emphasized. The differences between your proposed method and existing approaches need to be highlighted more clearly. I suggest strengthening the comparison in the introduction and discussion sections to explicitly outline the novel contributions of your work.

**Author response:** Thank you for your valuable feedback. In response to your comment, we have revised the seventh paragraph of the “Introduction” and added a new “Discussion” section that explicitly highlights the differences between our method and the current state-of-the-art, emphasizing our work's novel contributions.

We hope these changes address your concerns and clarify the innovation presented in our study. Thank you again for your insightful suggestions.

**Reviewer#2, Concern#3:** Your manuscript generally aligns with the journal’s scope, which covers topics related to mechatronic engineering. However, the experimental validation lacks strong supporting evidence. The manuscript would benefit from additional comparative experiments or a more detailed performance analysis. To improve the scientific rigor of the study, I strongly recommend providing more experimental data, especially direct comparisons with existing methods.

**Author response:** Thank you very much for your constructive feedback. We fully agree with your comment that the experimental validation would benefit from more comprehensive evidence and detailed comparisons.

In response, we have enriched the content of the subsection “***Comparison With the State-of-the-Art Methods***” by adding new experimental results. The newly added content is as follows:

“*To more intuitively demonstrate the performance differences among various models under strong noise conditions, we selected SNR = -8 dB (high noise intensity and covers a comprehensive range of comparison methods) to plot their confusion matrices and t-SNE dimensionality reduction visualizations on the CWRU test set, as shown in Figures 10 and 11.*

Fig.10 The comparison of confusion matrices of different models on the CWRU test set under SNR = -8 dB.

*From the confusion matrices, it is evident that models such as DRSN, AMFCN, Mel-CNN, and IDRSN have significant classification errors across multiple categories, primarily between categories 1, 2, 5, and 8. This may be due to the limited noise resistance of these methods, which makes it difficult to effectively distinguish between these inherently similar fault patterns under high noise interference. In contrast, our model only has a few misclassifications on categories 0, 1, and 2, with most errors being the misidentification of category 2 as category 0. This phenomenon may stem from the substantial similarity between categories 0 and 2 in the feature space, which is more easily obscured by intense noise. It is worth noting that although the overall recognition accuracy of the proposed model is slightly lower than that of RDDAN, its misclassifications are mainly between adjacent categories and are relatively concentrated, which to some extent reflects its strong ability to model feature boundaries and maintain high fault recognition capabilities even in high-noise environments.*

Fig.11 The t-SNE visualization of feature distributions of different models on the CWRU test set under SNR = -8 dB.

*The t-SNE dimensionality reduction visualization results shown in Figure 11 further corroborate this conclusion. It is visible that DRSN, AMFCN, Mel-CNN, and IDRSN have significant overlapping regions among categories 1, 2, 5, and 8, indicating their difficulty in achieving adequate category distinction under strong noise. In contrast, the feature distributions extracted by our method and RDDAN are much clearer, with distinct boundaries between different categories and almost no overlap, which is highly consistent with the confusion matrix results. This further demonstrates the proposed model's superior feature representation capability and good inter-class separability under complex noise conditions.”*

Additionally, we would like to kindly inform you that the category-color correspondence in the t-SNE visualizations (Figure 11) follows the same order and labeling as the first subfigure of Figure 7, which may help you interpret the visualization better. Although not explicitly mentioned in the manuscript, we hope this clarifies the mapping of fault types.

We hope that these additional experiment results and detailed discussions sufficiently address your concern and strengthen the study's scientific rigor.
